# Salbutamol in the Management of Asthma: A Review

**DOI:** 10.3390/ijms232214207

**Published:** 2022-11-17

**Authors:** Lara Marques, Nuno Vale

**Affiliations:** 1 OncoPharma Research Group, Center for Health Technology and Services Research (CINTESIS), Rua Doutor Plácido da Costa, 4200-450 Porto, Portugal; 2 Faculty of Medicine, University of Coimbra, Azinhaga de Santa Comba, Celas, 3000-548 Coimbra, Portugal; 3 CINTESIS@RISE, Faculty of Medicine, University of Porto, Alameda Professor Hernâni Monteiro, 4200-319 Porto, Portugal; 4 Department of Community Medicine, Health Information and Decision (MEDCIDS), Faculty of Medicine, University of Porto, Rua Doutor Plácido da Costa, 4200-450 Porto, Portugal

**Keywords:** salbutamol, asthma treatment, short-acting β_2_-agonists, pharmacokinetics, adverse effects, efficacy, safety

## Abstract

Asthma is a common inflammatory disease of the lungs. The prevalence of asthma is increasing worldwide, and the tendency indicates that the number of asthma sufferers will soar in the coming years for several reasons, in particular, the lifestyles we have adopted that expose us to risk factors. Salbutamol is the first selective short-acting β_2_-agonist (SABA) used as an alternative reliever in the treatment of asthma. Its therapeutic effect is based on its potent smooth muscle relaxant properties, which allow the inhibition of bronchial smooth muscle contraction and subsequent bronchodilation. Salbutamol can be administered orally, intravenously (IV), intramuscularly (IM), subcutaneously, or by inhalation. For this reason, the pharmacokinetic (PK) parameters—absorption, distribution, metabolism, and elimination—are highly diverse and, consequently, the efficacy and adverse effects also differ between each formulation. Here, we review the pharmacological profile of different salbutamol formulations, focusing on their efficacy and adverse effects for its original application, asthma.

## 1. Asthma Overview

Asthma is a chronic heterogeneous disease of the lower airways characterized by inflammation and airway hyper-reactivity leading to episodes of wheezing, breathlessness, chest tightness, and coughing [1,2,3]. According to a Lancet commission, our concept of asthma is too simplified [4,5,6]. The pathophysiology of asthma is really rather complex, due to the large number of cells and cellular elements involved. Their phenotypic characteristics—including clinical features of the disease and their underlying mechanisms (endotype)—are complex and represent a variety of host–environment interactions [5].

Genome studies of asthmatic children and adults have identified an association between polymorphisms for IL33, IL1RL1/IL18R1, HLA-DQ, SMAR3, and IL2RB9 and the locus on chromosome 17q21, including the genes ZPBP2, GSDMB, and ORMDL3, which have an important role in epithelial barrier function, and innate and adaptative immune responses, contributing to asthma [7,8,9]. However, the cause of asthma remains unclear. Several risk factors in turn are identified: genetic predisposition (hereditability ranges between 35 and 95%), events in early life, such as low-birth weight and prematurity, airborne environmental exposures (tobacco smoke, pollutants, and ozone), and viral respiratory infections contribute to the risk of disease. Furthermore, asthma is more likely in people who have other allergic conditions. Recently, studies including microbiome, stress, chemical exposure, and dietary changes as risk factors have emerged [1,10,11].

The different types of asthma include allergic asthma, non-allergic asthma, adult-onset asthma, exercise-induced bronchoconstriction (EIB), occupational asthma, asthma-COPD overlap, and pediatric asthma [12,13]. The most prevalent one, allergic asthma, is triggered by allergens, whereas non-allergic is brought on by stressful situations, viral infections, and extreme weather. Adult-onset asthma is the term used to describe those situations when people only experience their first asthma symptoms as adults. EIB, also known as exercise-induced asthma, occurs when, in asthmatic patients, physical activity causes airways to constrict. Of note, EIB is also experienced in non-asthmatic patients. People who usually work around chemical fumes, dust, or other air irritants may develop occupational asthma. Simultaneous asthma and chronic obstructive pulmonary disease (COPD) are recognized as asthma-COPD overlap [12,13]. Furthermore, the Global Initiative for Asthma (GINA) [14] distinguishes two additional clinical asthma phenotypes: asthma with persistent airflow limitation and obesity-associated asthma, as obese patients are more predisposed to respiratory problems.

Regarding disease severity, the 2022 GINA guidelines [14] classify asthma into three categories: mild, moderate, and severe (Table 1). This diagnosis, according to GINA, is based on the identification of respiratory symptoms typical of asthma, such as wheezing, shortness of breath, coughing, chest tightness, or a limitation of expiratory airflow (assessed from the bronchodilator reversibility test or from others).

This long-term condition affects all age groups, in particular, the pediatric population, representing the most common medical emergency [5,15]. The global prevalence has increased, with higher incidence in developed countries than in developing countries [16]. Although still being debated, several theories have been proposed to justify the high incidence of this disease. First, it was assumed that only exposure to environmental factors (air pollutants, indoor allergens) contributed to increases in asthma [17]. Strachan [18] suggested the “hygiene hypothesis”, which argues that excessive hygiene in children has a negative impact on the immune system, leading to decreased resistance to these conditions. Later, Rook et al. [19] proposed that lack of exposure to non-pathogenic and commensal microorganisms may also explain the high number of asthma cases. Nonetheless, this number is probably underestimated in resource-poor countries, due to the lack of basic asthma medications and access to health care. Leynaert et al. [20] investigated gender differences in the incidence of allergic and non-allergic asthma in the general population. In the younger population, asthma prevalence is higher in boys than in girls; however, in adults, the incidence rate is around 20% higher in women than men. The increased frequency in boys is attributed in part to smaller airways relative to lung size when compared to young girls.

Although asthma continues to be a major source of illness and mortality worldwide, there is a diverse range of therapeutic options [4,5,21]. The goal of treatment is to attempt to reduce fatalities and hospitalizations, as well as control the disease by reducing symptoms, preventing exacerbations, and restoring lung function, ensuring a normal standard of living in these patients [14,22,23]. According to GINA [14], contemporary asthma treatments are based on symptom relief using combined therapies. Low-dose inhaled corticosteroids (ICSs) and formoterol is recommended as first-line therapy. Alternatively, a short-acting β_2_-agonist (SABA) reliever with ICS is prescribed, which constitutes a very effective treatment in reducing symptoms and the risk of exacerbations, hospitalizations, and fatalities. Providing an asthma action plan (AAP) is also one of the suggested strategies. This document outlines the daily management as well as how to recognize and deal with worsening symptoms [14,24]. The pharmacological approaches include SABAs, ICS, long-acting β_2_-agonists (LABAs), oral corticosteroids, leukotriene receptor antagonists, long-acting muscarinic antagonists (LAMAs), immunotherapy, and monoclonal antibodies [22,25].

## 2. Short-Acting β_2_-Agonists

SABAs represent a class of drugs that have been used to treat asthma for thousands of years, initially as a naturally occurring compound in Chinese herbal medicine [23]. Bronchoconstriction is one of the hallmarks of asthma; hence, β-agonists, or bronchodilators, have often been prescribed as first-line therapy for quick relief [26], especially in patients with mild asthma [23]. The effectiveness of SABAs when provided “as needed”, however, has led to their widespread abuse. Evidence has emerged associating increasing SABA use to asthma mortality [27,28,29] and risk of exacerbations [30]; thus, interest in this drug class has declined over the years [23]. The prescription of β-agonists results in poorly controlled asthma, though not necessarily due to their direct negative effects, but rather because patients prefer SABAs instead of regular ICS and LABAs. In fact, the rapid symptom relief gives the illusion that asthma is being treated, despite the fact that the inflammation of the airways is not addressed [26]. The regular use of SABA is connected with β_2_-receptor downregulation, loss of bronchodilator response, increased airway hyperresponsiveness, and increased airway inflammation [31,32].

As a result of new evidence indicating a higher risk of exacerbations with SABA monotherapy and its overuse, in 2019, GINA introduced the most significant change in asthma treatment: SABA-only treatment is no longer recommended [33]. Therefore, these drugs are currently used as alternative relievers, whereas ICS-containing treatment should be the first-line treatment for patients aged 12 years or older [14,23,33,34]. The mainstay of acute asthma therapy, however, remains bronchodilators, along with systemic corticosteroid therapy and controlled flow oxygen supplementation [14,35].

Notwithstanding current guidelines, clinical practice continues to be based on the excessive use of SABA relievers. Akker et al. [36] performed a retrospective analysis using medical records of adult asthmatic patients at a health center in the Netherlands. Of the total individuals under study, 25% overused SABAs and, among these patients, 19% experienced exacerbations. The authors concluded that clinicians still prescribe SABA as they are unaware of this problem.

Recently, a program—SABA Use IN Asthma (SABINA)—was developed to investigate the overreliance on SABAs worldwide and its impact on clinical outcomes [37]. The SABINA program, using real-world observational studies, confirmed the large number of asthmatic people who overuse SABA inhalers and confirmed that there is a link between high bronchodilator use and severe risk of exacerbations [30,36].

## 3. Salbutamol: A First Approach

Salbutamol, the first selective SABA extensively used in clinical practice, was introduced in 1968 [38]. It is a selective β_2_-adrenergic receptor agonist used for acute episodes of bronchospasm caused by asthma as well as other chronic bronchopulmonary disorders [39]. It is indicated for the symptomatic relief and prevention of bronchospasm due to its potent smooth muscle relaxant properties [40,41]. The World Health Organization (WHO) ranks salbutamol as one of the most effective and safest medicines essential to healthcare systems [42]. It is associated with improved daytime symptoms, although a subtle deterioration in asthma control may occur over time [43,44]. Salbutamol monotherapy is not indicated, as we previously stated. It is recommended either in combination with ICS or as an alternative approach in specific conditions. In fact, this drug may have a pro-inflammatory effect when administered regularly, according to Gavreau et al. [45], which may explain the higher risk of exacerbations that has been reported. This evidence is supported by Ritchie et al. [46]. The authors claim that β-agonists increase the number of inflammatory mediators, which leads to airway obstruction and hyperresponsiveness, allowing speculation that excessive use of bronchodilators may cause an exacerbation.

### 3.1. Chemistry

**Figure 1:**
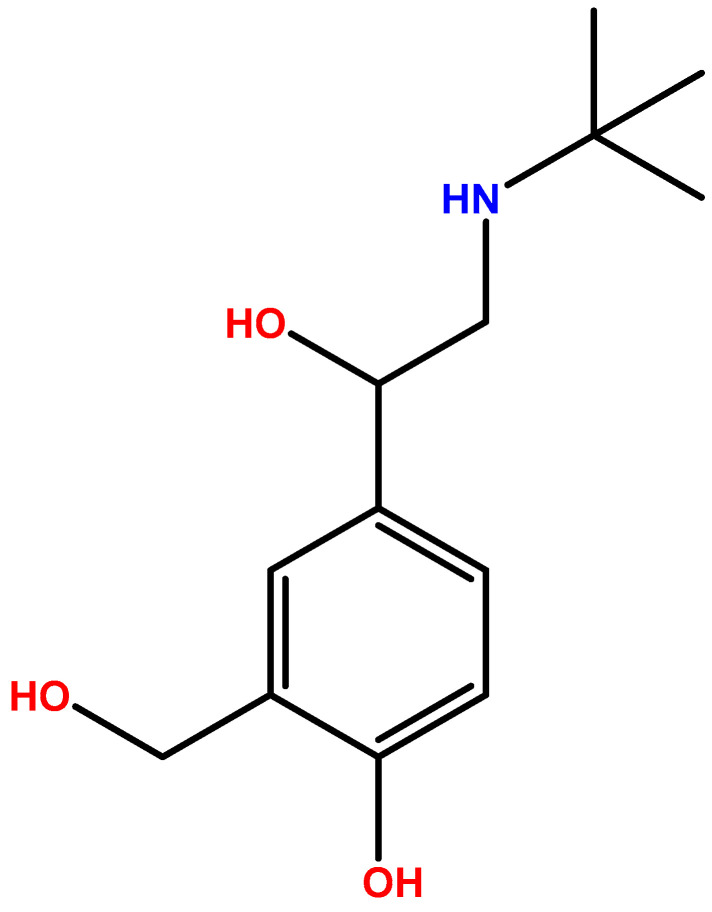
Chemical structure of salbutamol.

Salbutamol (Figure 1) is a chiral drug with (R)- and (S)-isomers [47]. Its pharmacological activity is associated to the (R)-enantiomer because it binds to the human β_2_-adrenoceptor. The activity of the (S)-enantiomer is controversial [4,47,48,49,50]. Although this isomer is assumed to be inert in humans, Patel et al. [47] reported an experimental study that suggested that (S)-isomer may have clinically significant adverse effects. Furthermore, it is believed that (R)-salbutamol in its non-racemic form has beneficial effects. Gumbhir-Shah et al. [51] reported identical PK, pharmacodynamics (PD), and safety of this isomer, provided either as the single enantiomer or racemic mixture by inhalation to subjects with mild to moderate asthma. Controversially, several well-conducted studies reveal that this isomer is not clinically superior to racemic salbutamol [47].

### 3.2. Pharmacokinetics and Metabolism

The pharmacokinetics (PK) of salbutamol depends on many variables. The formulations and the delivery mechanism (MDI or DPI) used have an impact on the amount of drug that reaches the airways, absorption, and, consequently, effectiveness and the side effect profile [52]. Following inhalation, the systemic levels of salbutamol are undetectable, since it first acts topically on bronchial smooth muscle [39]. After 2–3 h, low plasma concentrations are observed due to the swallowing and oral handling of the inhaled drug. Oral administration is rapidly and well absorbed, with peak plasma salbutamol concentration observed after 2 h. However, the drug undergoes the first-pass effect, related to both strong hepatic and presystemic metabolism in the intestinal mucosa, resulting in only 50% of bioavailability [52]. The majority of data on salbutamol blood and urinary concentrations come from studies on healthy non-asthmatic participants who have never taken SABAs [53]. However, as Elers et al. [53] concluded, PK of inhaled and oral salbutamol did not differ between β_2_-agonist-naïve non-asthmatic subjects and asthmatic individuals using regular anti-asthmatic medication. Lewis et al. [54] studied 11 acute severe asthmatic patients and all of them presented low or undetectable plasma concentrations of salbutamol after inhalation treatment. There are few PK studies on salbutamol provided intravenously (IV) [40].

Salbutamol is mainly metabolized by sulfate conjugation into the 4′-O-sulphate ester, which possesses negligible pharmacologic activity [39]. This occurs in the liver, where the metabolizer enzyme sulfotransferase is found [55]. The metabolism can also occur in the gastrointestinal tract, due to the swallowing of an inhaled dose, and in the cytochrome P540 enzyme system (minor metabolic route) [39,56,57]. As aforementioned, this β_2_-agonist is not totally absorbed after inhalation or oral administration, resulting in about 30% of non-metabolized drug. In turn, the portion of non-metabolized salbutamol is approximately 65% when it is administered intravenously. Following metabolization, most of the intake drug is excreted in the urine within 24 h, with a small fraction eliminated in the feces [39]. The elimination of (R)-salbutamol is substantially faster than that of (S)-salbutamol, since the latter is metabolized up to 10 times slower than (R)-salbutamol [40,58]. Some theories on the subject have been proposed. Both isomers may have different metabolism pathways; however, Ward et al. [59] revealed that there was no differential lung metabolism between (R)- and (S)-salbutamol. The elimination half-life of inhaled or oral salbutamol has been recorded as being between 2.7 and 5 h while after intravenous (IV) administration it has been documented as being approximately 3–4 h [38,39]. Clearance is reported to be 272 ± 38 mL/min after oral administration and 291 ± 70 mL/min after an IV administration [39]. The similarity between oral and inhaled excretion patterns assumes the presupposed theory: a significant portion of an inhaled dose is swallowed [60].

### 3.3. Mechanism of Action

**Figure 2:**
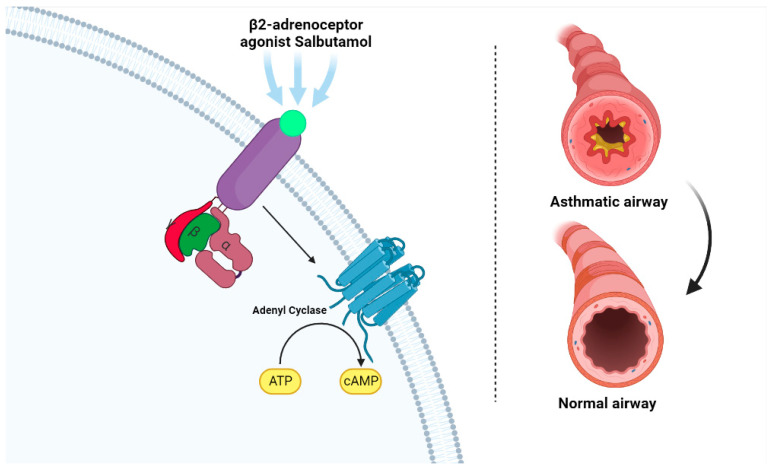
Mechanism of action of salbutamol (**left**). The β_2_-adreneceptor agonist (light green) binds to the β_2_ receptor (purple), which activates adenyl cyclase, resulting in the conversion of ATP to cyclic AMP (cAMP). This promotes bronchodilation and relieves symptoms experienced during an acute asthma episode (**right**). Created with Biorender.com. Available online: http://biorender.com/ (accessed on 11 October 2022).

The smooth muscle of the respiratory tract is constituted by a large number of β_2_-receptors. Their activity is mediated by the production of cyclic adenosine monophosphate (AMP) as a second messenger. Therefore, as an agonist, salbutamol binds reversibly to these receptors, which are believed to be adenyl cyclase, resulting in the conversion of cyclic AMP (Figure 2). Cyclic AMP then triggers a cascade of intracellular events that culminate in the inhibition of the contraction of bronchial smooth muscle, thereby promoting smooth muscle relaxation and bronchodilation—its therapeutic effect. Salbutamol also inhibits the release of immediate hypersensitivity mediators from cells, particularly mast cells. Due to its high selectivity, salbutamol has minimal activity on β_1_-adrenergic receptors [61,62,63,64].

### 3.4. Pharmacodynamic Properties

The major key physiological role of salbutamol is its bronchodilator effect in the lungs [65,66,67]; however, it also possesses other properties, including cardiovascular, uterine, metabolic, and neurological effects. Usual therapeutic doses of inhaled salbutamol do not significantly affect the cardiovascular system, unlike other formulations [64]. A study in healthy volunteers revealed that IV or nebulized salbutamol induced a dose-related increase in heart rate and systolic blood pressure [68,69]. In asthmatic patients, inhaled and oral salbutamol raised heart rates by 23% and 28%, respectively [70,71]. However, this increase is observed as well in asthmatic patients with cardiovascular disease [64], highlighting the caution with which these patients should be treated when they are prescribed salbutamol. On the other hand, in patients with chronic heart failure, there are beneficial effects when treated with this β_2_-agonist [72].

Salbutamol decreases potassium concentration in blood. The mechanism underlying this process is assumed to be related to the stimulation of β-adrenoceptors linked to membrane-bound Na/K ATPase on skeletal muscle, which induces an influx of potassium into cells and a subsequent reduction of plasma potassium concentration [73,74]. The lipid effects of this drug are identified as increased blood levels of non-esterified fatty acid (NEFA), insulin, and HDL-cholesterol [75,76,77].

Wager et al. [75] investigated the cardiovascular and metabolic effects of oral and IV salbutamol in diabetic and non-diabetic pregnant women throughout the third trimester. The findings showed a significant increase in plasma levels of insulin, carbohydrate, and lipid metabolites, revealing that glycogenolysis, lipolysis, and insulin secretion were stimulated. Diabetic women have more pronounced glycogenolytic and lipolytic effects, due to their impaired insulin function. For this reason, salbutamol should be carefully prescribed to diabetics. Rolf Smith and Kendall [74] also stated the association between β-receptors and glycogenolysis, and insulin release, with their satisfactory results in increasing plasma glucose and insulin concentrations in healthy volunteers.

Salbutamol is thought to possess antidepressant properties. Although the clinical relevance of these findings is unknown and this topic is still underexplored in the scientific community, it has been proposed that these benefits are mediated through an increase in serotonergic system activity [78].

Pregnancy is not affected by this β_2_-agonist drug. A study designed to assess the effect of long-term high-dose oral therapy with salbutamol in previous multiple pregnant women showed no effect of salbutamol on current pregnancy duration nor birth weight [79]. Nevertheless, since it may enter the embryo through the placenta, it is likely to have an impact on the fetus’ metabolism, despite the scarcity of human research in this area [64].

Due to its inhibition of mast cell mediator release in asthmatic patients, reducing changes in forced expiratory volume (FEV), plasma histamine, and neutrophil chemotactic activity (NCA), salbutamol has an impact, albeit minimal, in inhibiting allergic responses [64]. This β_2_-agonist may be a potent drug for treatment of multiple sclerosis (MS), due to its ability to regulate the expression of several cytokines [38]; however, this topic has not yet been explored. As far as MS-related fatigue is concerned, Almeida et al. [80] questioned the possibility of using salbutamol as an alternative treatment. In a group of 30 patients with relapsing-remitting MS and fatigue, treatment with this SABA did not improve this condition.

### 3.5. Adverse Effects

β_2_-adrenergic receptors, in addition to being found in lung membranes, are found in skeletal vascular, liver, and cell membranes [81]. Salbutamol may thus have effects other than the bronchodilator effect that has been reported. Furthermore, since salbutamol is available in a variety of dosage forms, the side effects are also rather diverse. In fact, IV is the route of administration with the greatest adverse effects identified, followed by oral and nebulized administrations. The inhaled form represents the safest route of administration [64].

The musculoskeletal system might be impacted at the tremor level, as well as at myopathy. Based on a case report of a 76-year-old asthmatic woman, Hellier et al. [82] investigated the possibility of salbutamol causing myopathy. The authors concluded that salbutamol may be responsible for the deleterious muscle effects, and they proposed that β_2_-adrenergic receptors be added to the list of potentially myopathy-inducing drugs. Notwithstanding, salbutamol has been proven to influence skeletal muscle strength in young men. A clinical trial aimed to determine the impact of daily administration of a sustained-release salbutamol formulation (8 mg via oral administration) on skeletal muscle functional capacity, and the authors found that salbutamol boosted skeletal muscle function [83]. Due to these reported ergogenic effects, in addition to its bronchodilator effect, some athletes have used salbutamol to improve their physical performance [84]. Therefore, since 2004, salbutamol has been included in the List of Prohibited Substances and Methods of the World Anti-Doping Agency (WADA) [85]. It has been proven that only systemic salbutamol influences physical performance, whereas inhaled salbutamol at therapeutic doses has no significant effect, which may be explained by insufficient systemic exposure. For this reason, only inhalation administration is allowed, but the dose should not exceed 1600 μg per day (equivalent to twice the maximum recommended daily dose) [84,85]. The rules proposed by WADA in 2022 specify a maximum of 1600 μg over 24 h in divided doses, not to exceed 600 μg over 8 h starting from any dose [86].

The cardiovascular side effects of bronchodilators are one of the major concerns. In particular, salbutamol may cause tachycardia and peripheral cardiac vasodilation-induced reflex [87,88]. Tachycardia, although it is not a threat to patients’ health, is mainly caused when salbutamol is administered via an inhaler. Investigations on the subject have shown that tachycardia is caused by the inhaled portion rather than the swallowed fraction [89]. Several studies report arrhythmias and angina after salbutamol administration. Patients with severe hypoxemia and low serum potassium have an increased risk of developing cardiac dysrhythmias [89]. Asthmatic patients who have concomitant cardiovascular disorders (including coronary insufficiency, cardiac arrhythmias, and hypertension) should use this SABA carefully, due to an increased risk of developing severe cardiac adverse effects [64]. Nevertheless, compared to non-selective β-agonists, it exhibits reduced β_1_-mediated cardiac adverse effects, since it is a selective β_2_-agonist [90]. Actually, a comparison study of the incidence and type of cardiac arrhythmias demonstrated that controlled-release salbutamol has no significant detrimental effects on cardiac rhythm [91]. A pharmacovigilance study performed by Sato et al. [91] correlated the use of salbutamol and the incidence of Takotsubo syndrome (TTS), a reversible left ventricular systolic dysfunction.

The respiratory system may also be impacted. Some patients have experienced a feeling of “thick neck”, chest heaviness, erythema, and pulmonary edema [92,93]. Paradoxical bronchoconstriction may occur in rare asthmatic cases. This reaction has been reported in some case reports: patients with historical asthma develop an airway obstruction after subsequent doses of salbutamol (inhaled or nebulized) [94,95]. This SABA drug is used (in recommended doses) in bronchodilator tests in elderly patients who have a higher risk of respiratory diseases owing to repeated lifelong exposure to environmental toxins [96].

At the metabolic level, salbutamol can cause hypokalemia and increases in insulin, glucose, pyruvate, free fatty acids (FFA), and lactate. A study was conducted to investigate the plasma glucose concentrations in patients receiving salbutamol subcutaneously, intramuscularly (IM), or intravenously (IV) [97]. The results showed substantial increases in all three treatment groups, although patients who received salbutamol IM and subcutaneously have a more pronounced rise. Torella et al. also explored the effect of IV salbutamol on some metabolic and hormonal parameters in both healthy and diabetic subjects. They found an increase in blood sugar levels, FFA, and insulin, which suggests that salbutamol should be cautiously used for diabetic patients [98]. Another noteworthy study was carried out to assess the serum phosphate levels following the administration of nebulized salbutamol during the emergency treatment of acute asthma exacerbation, and the results showed no statistically significant reduction in serum phosphate [99]. Several case reports have recently been published of asthmatic patients who took salbutamol and experienced an increase in lactate levels, a condition known as salbutamol-induced lactic acidosis [100,101,102].

Hypokalemia is identified as a metabolic side effect of salbutamol, particularly at high doses of salbutamol IV and nebulizer [103,104,105]. Due to the lowered potassium level effect, this drug has been used to treat acute hyperkalemia by reducing the plasma potassium concentration [106]. It has been used successfully in the nebulized form in neonates. Murdoch et al. [107] also studied IV salbutamol for the treatment of hyperkalemia in children and observed a reduction in the mean plasma potassium concentrations. The risk of hypokalemia is greater when salbutamol is provided simultaneously with corticosteroids [108] and theophylline [109].

In the nervous system, salbutamol may cause hallucinations, tremors, and anxiousness [110,111,112]. This can be explained by the ease of salbutamol crossing the blood–brain barrier (BBB). Tremors are believed to be triggered by an imbalance between twitching muscle groups in the limbs rather than by CNS stimulation. Seizures have been recorded when patients, especially young ones, overuse salbutamol inhalers [113]. Additionally, it is often assumed that using salbutamol to treat asthma episodes causes seizures in epileptic and asthmatic patients. Uysalol et al. [114] designed a study to understand the relationship between salbutamol and seizures in patients aged 2–18 years with asthma and epilepsy. Contrary to popular opinion, the results for the use of salbutamol were positive: the seizure rate was higher in the group of patients who did not take this drug.

Parkinson’s disease (PD) is also associated with salbutamol administration. However, no significance in reduced risk of PD was revealed by a meta-analysis developed by Singh et al. [115] with the purpose of examining the association between β-adrenergic drugs’ use and PD.

Regarding tumorigenicity, although it has been observed in animals, salbutamol does not induce cancer development in men. The authors claim that such findings are irrelevant to humans since animal exposure levels during toxicity studies are greater than those ever prescribed to men [63,116].

The several adverse effects of salbutamol are well reported. SABA overreliance is the main reason for this large number of occurrences. As previously mentioned, when patients experience a worsening of symptoms, they tend to increase their use of SABA, leading to a greater risk of adverse outcomes. Nevertheless, salbutamol is part of the Essential List of Medicines provided by the WHO [42], and is considered “one of the safest and most effective drugs currently available”.

### 3.6. Clinical Efficacy

Salbutamol has been used successfully in the management of severe acute asthma, constituting the standard emergency treatment for symptom relief and for the treatment of childhood asthma [64]. Since children show a faster and more complete response to bronchodilators than adults, salbutamol constitutes the first-line treatment for all asthmatic children [64,117]. Children, in fact, receive much higher doses of IV salbutamol per kg of weight than adults [118]. In addition, those responses are complemented by a significantly low incidence of side effects when compared to adults, demonstrating a high level of tolerance [117].

There are several comparison effectiveness and safety studies of salbutamol and other bronchodilators as well as other asthma-class drugs. Salbutamol is more effective than isoprenaline, a non-selective β-adrenoreceptor agonist, and isoetarine, a selective β-adrenoreceptor agonist. Otherwise, it is quite similar to bitolterol, broxaterol, clenbuterol, fenoterol, metaproterenol, procaterol, terbutaline, and tulobuterol (all bronchodilators) from a clinical point of view [64]. A clinical trial examined the bronchodilator response in adults with stable asthma following salbutamol and formoterol administration [119]. The group of patients who took salbutamol showed a higher clinical response (forced expiratory volume, FEV). The efficacy and safety of levalbuterol (SABA with the more active R-enantiomer of salbutamol racemic mixture) compared to salbutamol have also been discussed. Jat et al. [120] consider that levalbuterol is not clinically superior and should not be used over salbutamol for the treatment of acute asthma. According to a randomized placebo-controlled trial [121], no significant differences between levalbuterol and salbutamol in terms of FEV were documented. Regarding the comparison between IV salbutamol and IV aminophylline, it was observed that, in the doses and in the routes of administration targeted in the study, salbutamol was equally effective compared to aminophylline [122].

When comparing salbutamol and salmeterol, salmeterol has a prolonged bronchodilator effect in healthy adult volunteers [123]. A similar statement was made for asthmatic patients: a comparative study of the dry powder formulations of salmeterol and salbutamol revealed that salmeterol is more successful in managing asthma [124]. Likewise, a prolonged protective effect of salmeterol is observed. In patients with exercise-induced asthma, inhaled salmeterol exhibited a long-lasting effect, related to the compound’s lipophilic property, which is responsible for the slow clearance of the molecules from the system [125]. None of these drugs were associated with a worsening of the disease. However, a subsensitivity to salbutamol’s bronchodilator effects induced by regular treatment with salmeterol in patients with asthma was reported [126,127].

The interaction of salbutamol with other drugs has also been explored. It is usually combined with ICSs, such as budesonide, fluticasone, and mometasone. In a randomized double-blind two-period single-dose crossover study [128], the authors sought to assess the efficacy and safety of salbutamol–budesonide compared with placebo in patients with asthma and EIB. Adolescents and adults who took this drug combination approximately 30 min before exercise displayed a more effective symptom relief than placebo. Additionally, Papi et al. [129] supervised a clinical trial that compared the efficacy and safety of these two drugs. It was demonstrated that salbutamol alone increases the risk of severe asthma exacerbation, suggesting that combining salbutamol with budesonide is a better treatment strategy. Indeed, the beneficial effects of this combination were proven in more than one study: another clinical trial [130] confirmed that such a combination improves lung functions as well as anti-inflammatory and anti-allergic effects in patients with acute bronchial asthma. Siddiqui et al. [130] have also studied the use of this SABA in conjunction with magnesium sulfate (MgSO_4_) in the management of acute asthma in Indian children. The authors concluded that the addition of nebulized MgSO_4_ to salbutamol did not enhance lung function. The findings of the clinical trial conducted by Sarhan et al. [131], on the other hand, were rather different. They concluded that nebulized MgSO_4_ with salbutamol has a considerable bronchodilator effect, suggesting this form of treatment may be the best choice for the management of acute asthma exacerbations.

Increasing doses of salbutamol to salmeterol was the focus of the study published by Smyth et al. [132]. The data suggest that this interaction does not alter the beneficial or adverse effects of salbutamol in patients taking salmeterol.

A double-blind placebo-controlled study [133] was conducted with the purpose of uncovering the differences in the bronchodilator effects of salbutamol with or without sulfate in asthmatic patients. No clinically significant difference was found. Additionally, a therapy based on a combination of salbutamol and beclomethasone dipropionate improves asthma control more than increasing the dose of salbutamol [134]. To examine the bronchodilator effects of three sets of treatments—salbutamol combined with oxitropium bromide, a low salbutamol dose, and a high salbutamol dose—Laitinen et al. [135] conducted a controlled trial on adult asthmatic patients. Both the combination and high salbutamol dosage therapies showed more effectiveness than the low salbutamol dose treatment.

Salbutamol administered with ipatropium bromide was the subject of a prospective randomized double-blind study in children aged 2–18 years with severe to moderate asthma [136]. The goal was to determine if this drug combination impacted oxygenation, lung function, and the number of hospitalizations. In fact, there was a significant decrease in hospitalizations and an improvement in lung function in all children, particularly pronounced in children with severe asthma attacks. The therapeutic approach of combining nebulized salbutamol with salbutamol–ipatropium bromide has also been proven to be beneficial for treating acute asthma attacks in patients with moderate asthma [137].

Another therapeutic approach in asthmatic children is to associate salbutamol and theophylline. A double-blind randomized controlled trial [138] demonstrated that these drugs interact negatively, resulting in the occurrence of tachycardia.

In addition to the aforementioned drug interactions, salbutamol has many other interactions; for example, with β-blockers (antagonists) [139], corticosteroids [108], and diuretics [140].

### 3.7. Routes of Administration

**Figure 3:**
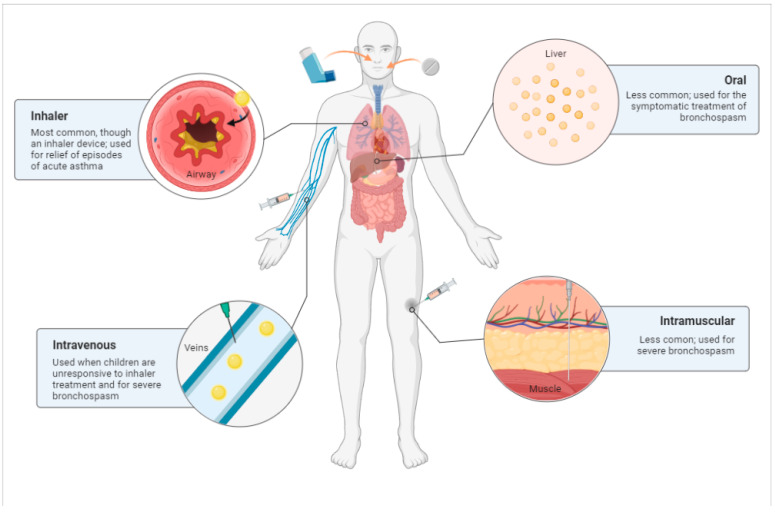
Main routes of administration of salbutamol: inhalation, oral, intravenous, and intramuscular. Created with BioRender.com. Available online: http://biorender.com/ (accessed on 11 October 2022).

Salbutamol can be administered intravenously, intramuscularly, subcutaneously, via inhalation, or orally (Figure 3). Due to its greater efficacy at low doses, the inhalation route is the administration currently employed in daily practice. The dosages required, efficacy, and adverse effects differ significantly among these routes [38,57].

IV salbutamol is used as second- or third-line treatment for severe acute asthma [40]. Palpitations, tremor, and postural hypotension occur when the drug is injected IV [141]. Rebuck et al. argued that IV administration was associated with increases and decreases in heart rate and diastolic blood pressure [64]. Increased glucose and insulin concentrations as well as decreased plasma potassium concentrations are also documented [98,103,104,105]. These side effects may be caused, in part, by the scarcity of PK data on IV salbutamol. Dosing recommendations are not based on well-conducted PK and PD studies [142,143,144]. In turn, IM salbutamol is prescribed for the emergency treatment of asthma. No statistically significant differences were found between the bronchodilator effect of IV and IM doses of salbutamol in asthmatic patients [145].

Inhaler salbutamol can be administered via a nebulizer or a spacer/inhaler. Nebulization constitutes the inhalation of a wet aerosol, and it is recommended for the management of acute asthma in children [64,146]. It has a protective effect on airways in children under one year old [147]. This type of administration typically requires higher doses and, as a result, dose-related adverse effects are reported [64]. In fact, normal volunteers who received the guideline therapeutic doses of nebulized salbutamol experienced significant increases in heart rate and systolic blood pressure, as well as significant decreases in diastolic blood pressure and serum potassium when compared to placebo [69]. Furthermore, patients with asthma frequently misuse inhaler devices, which can have a negative impact on clinical outcomes [148].

The first-line treatment is the inhalation of salbutamol via spacer/inhaler. It is characterized by its rapid onset, low frequency of adverse effects, and convenience of administration [43,149]. Salbutamol is prescribed every 20 min in the initial treatment, at a dose of 0.05 to 0.15 mg/kg. Afterward, the dose can be increased to 0.45 mg/kg, with a maximum dose of 20 mg per hour [150,151]. Tukiainen and Terho [152] investigated the short-term bronchodilator effects of dry powder and pressurized aerosol and concluded that this kind of administration is an effective method of drug delivery to the lungs, even in asthmatic patients with poor ventilation. Studies comparing the two routes of inhalation revealed no differences in treatment efficacy [65,153,154]. Thus, inhaled salbutamol is considered the best option for patients with reversible obstructive airway disease, with bronchodilation occurring 10 min after drug administration and lung function improving for up to 6 h. However, mild skeletal muscle tremors and cardiovascular-related effects have been reported [64].

The controlled release of salbutamol tablets in asthmatic patients has been demonstrated. Oral treatment is usually combined with inhalation of salbutamol [155]. The combination therapy has superior therapeutic effects compared to the inhalation treatment alone, according to a double-blind placebo-controlled trial. Therefore, it is highly helpful for children able to inhale from a powder device and swallow tablets. It is advised that children aged between 2 and 6 years take salbutamol syrup [156,157,158]. However, oral salbutamol is not recommended during an asthma exacerbation since the effects are not superior to inhaled preparations, have a slower onset of action, and have a higher frequency of side effects [35].

Grimwood et al. [159] compared three different methods of administering salbutamol—tablets, inhalational powder, and nebulizer. They stated that nebulized salbutamol has the maximal bronchodilatation effect. However, due to its high cost, it was once only recommended for patients with severe asthma. In less severe cases, the combined therapy of powder and tablet (easily administered and with a rapid onset of action) was prescribed [159,160,161]. Of note, these routes of administration have since been discontinued. Current clinical practice is based on inhaled administration.

Higher doses of salbutamol have been used in patients who are unresponsive to standard treatment. However, this results in the intensification of adverse effects [150]. The recommended dosages for salbutamol formulations are shown in Table 2.

## 4. Future Perspectives

Salbutamol has been used for over 50 years. To date, other drug families have been approved and new and increasingly effective drug-delivery devices have been designed. However, disease control is far from perfection: the proportion of uncontrolled asthma patients remains steady. The future of asthma is undoubtedly tough [10,162,163,164].

The European Asthma Research and Innovation Partnership (EARIP) [164] has defined 15 research priorities and its members deeply believe that if these are addressed, asthma deaths and exacerbations will reduce and patients’ quality of life will improve. Among them is precision medicine, which plays an active role in this disease. Targeting the right treatments to the right patients at the right time may be the key to improving therapeutic effectiveness.

Adherence is the most real obstacle to drug efficiency, according to healthcare professionals [162]. This is largely due to incorrect assumptions and fears about medications and drug-delivery devices. Since most of these drugs are available in inhaler devices, the embarrassment of using them in public and the side effects’ occurrence limit their use. Indeed, 60–70% of asthmatic patients admit not following their prescription [10]. During the COVID-19 pandemic, the problem of adherence to therapy was more evident [165]. Thus, it is critical to make people aware in order to change their beliefs and concerns and, consequently, their behavior towards the asthma disease. People must be educated about the safety and efficacy of asthma drugs, the proper manner in which to use inhaler devices, and about the worrying statistics related to this disease.

Asthma is an incredibly heterogeneous disease. The majority of medical treatments, including the prescription of salbutamol, are designed for the “typical patient”. This method may be successful for some patients but not for others. The Lancet commission, sharing this viewpoint, believes that efforts should be made to comprehend the pathophysiology of each patient more thoroughly [4]. One of the efforts that may be accomplished is research into different asthma phenotypes and stratification approaches, as well as a better understanding of these phenotypes [163,164]. Additionally, despite SABA monotherapy—namely, salbutamol therapy—not being included in the future of asthma management, asthmatic patients still prefer these drugs, according to statistics discussed in this review. Also, it continues to be recommended as an alternative reliever or in combination with ICS; therefore, future studies on this topic are important.

In silico modeling is a trending strategy that could be a boost to precision medicine, mainly due to its contributions to the improvement of drugs’ PK and PD knowledge. In fact, the PK information of salbutamol, in particular, is relatively limited and quite old, demanding the development of studies to collect current data on the drug’s behavior and its absorption, distribution, and elimination in asthmatic patients, according to its characteristics. Likewise, there are no pharmacological simulation studies of this specific drug. These studies allow for the prediction of the interaction between the drug, the disease, and the patient and, from the standpoint of precision medicine, they are relevant because the simulation may be confined to a group with specific traits, or else solely to a patient.

## Figures and Tables

**Table 1:** The classification of asthma severity according to the 2022 GINA guidelines; Adapted from [14].

Severity Level	Clinical Characteristics
Mild asthma	Controlled using as-needed ICS-formoterol, or with low dose ICS with as-needed SABA
Moderate asthma	Controlled with low- or medium-dose ICS-LABA
Severe asthma	Requires high-dose ICS-LABA to prevent it from becoming uncontrollable, or asthma that is still uncontrolled despite this treatment

**Table 2:** Recommended dosages for salbutamol formulations..

Clinical Use	Inhaler (100 μg)	Dry Powder Inhaler (200 μg)	Nebulizer (5 mg/mL)	Oral Syrup (2 mg/5 mL)	Oral Tablets (2 or 4 mg)	Intramuscular Subcutaneous	Intravenous
Intermittent asthma attacks or acute bronchospasm	A: 1 to 2 puffs every 4 h up to 4 times a day C: 1 puff every 4 h up to 4 times a day	1 puff up to 4 times per day	AC: 0.5 to 1 mL	A: 5 mL to 20 mL, up to 4 times a day C: 2.5 or 5 mL, 3 or 4 times a day	A: 4 mg, 3 or 4 times a day C: 1 or 2 mg, 3 or 4 times a day	A: 500 μg every 4 h	A: 250 μg injected slowly
Exercise-induced bronchoconstriction	A: 2 puffs 15 min before exercise C: 1 puff 15 min before exercise	1 puff 10 to 15 min before exercise	NA	NA	NA	NA	NA
Continuous treatment	NA	NA	1 to 2 mg per hour	NA	NA	NA	NA

A—adults; C—children; NA—not applicable.

## Data Availability

Not applicable.
